# Nano-Based Theranostic Tools for the Detection and Elimination of Senescent Cells

**DOI:** 10.3390/cells9122659

**Published:** 2020-12-10

**Authors:** Jagoda Adamczyk-Grochala, Anna Lewinska

**Affiliations:** Department of Biotechnology, Institute of Biology and Biotechnology, University of Rzeszow, Pigonia 1, 35-310 Rzeszow, Poland

**Keywords:** nanomaterials, cellular senescence, senotherapy, senolytics, nanotherapeutic-mediated senolysis

## Abstract

The progressive accumulation of apoptosis-resistant and secretory active senescent cells (SCs) in animal and human aged tissues may limit lifespan and healthspan and lead to age-related diseases such as cancer, neurodegenerative disorders, and metabolic syndrome. Thus, SCs are suggested targets in anti-aging therapy. In the last two decades, a number of nanomaterials have gained much attention as innovative tools in theranostic applications due to their unique properties improving target visualization, drug and gene delivery, controlled drug release, effective diagnosis, and successful therapy. Although the healthcare industry has focused on a plethora of applications of nanomaterials, it remains elusive how nanomaterials may modulate cellular senescence, a hallmark of aging. In this review paper, we consider novel nanotechnology-based strategies for healthspan promotion and the prevention of age-related dysfunctions that are based on the delivery of therapeutic compounds capable to preferentially killing SCs (nano-senolytics) and/or modulating a proinflammatory secretome (nano-senomorphics/nano-senostatics). Recent examples of SC-targeted nanomaterials and the mechanisms underlying different aspects of the nanomaterial-mediated senolysis are presented and discussed.

## 1. Cellular Senescence

Cellular senescence (CS) is characterized by a state of permanent cell growth arrest with altered metabolic features (e.g., affected glycolysis, mitochondrial function, and autophagic flux), modified protein secretion, and biomolecular damage that occurs in response to stressful stimuli [1,2]. CS was originally described in 1961 by Hayflick and colleagues, who observed that normal diploid embryonic fibroblasts had a limited ability to proliferate in an in vitro culture [3]. Based on these results, Hayflick coined the term replicative senescence to describe this phenomenon. Subsequently, progressive telomere shortening promoting DNA damage response (DDR) has been recognized as one of the major determinants of replicative senescence [4,5]. Later, the presence of senescent cells (SCs) in aging tissues that linked cellular senescence with organismal aging was documented [6]. Cells can also prematurely activate the senescence program upon treatment with stress stimuli such as UV radiation, oxidants, or DNA-damaging agents [7,8]. This process is known as stress-induced premature senescence (SIPS) [9]. Moreover, the expression of oncogenes could trigger senescence prematurely, in a process called oncogene-induced senescence (OIS) [10]. Cellular senescence may be considered as a tumor suppression mechanism by blocking the proliferation and division of cells with unrepaired DNA damage to prevent their stepwise malignant propagation [11]. However, it has also been demonstrated that cellular senescence may participate in other physiological processes such as specific tissue remodeling (so-called developmentally-programmed senescence) that acts in a damage-independent manner during embryonic development and wound healing in adulthood [12,13]. Despite the protective role of cellular senescence during stress-induced cellular responses, it is commonly accepted that CS may promote aging and contribute to the development of age-related diseases. Namely, it has been revealed that diverse pathological conditions associated with accelerated aging such as cancer, neurodegenerative diseases, cardiovascular disorders, and progeroid and metabolic syndromes are accompanied by the accumulation of SCs and cellular senescence may be a causative factor for the progression of age-related pathologies [14,15,16,17,18,19].

Many different molecular pathways have been proven to contribute to cellular senescence [20,21]. However, one of common features of all SCs is irreversible cell-cycle arrest regulated by p53/p21^WAF1/Cip1^ and p16^INK4A^/RB (retinoblastoma) tumor-suppressor pathways, which can interact with each other or act independently [2]. Unlike quiescent cells (the cells at the G0 phase) or terminally-differentiated cells, SCs do not respond to growth and mitogenic stimuli, thus they are irreversibly withdrawn from the cell cycle [17,22]. Two cell-cycle inhibitors that are often overexpressed in SCs, namely p21^WAF1/Cip1^ (encoded by *CDKN1a* gene) and p16^INK4A^ (encoded by *CDKN2a* gene) are crucial mediators of senescence. Accumulation of these cyclin-dependent kinase inhibitors (CDKI) resulted in permanent activation of RB family proteins, inhibition of E2F family transcription factor activity, and finally cell-cycle arrest [20].

**Figure 1:**
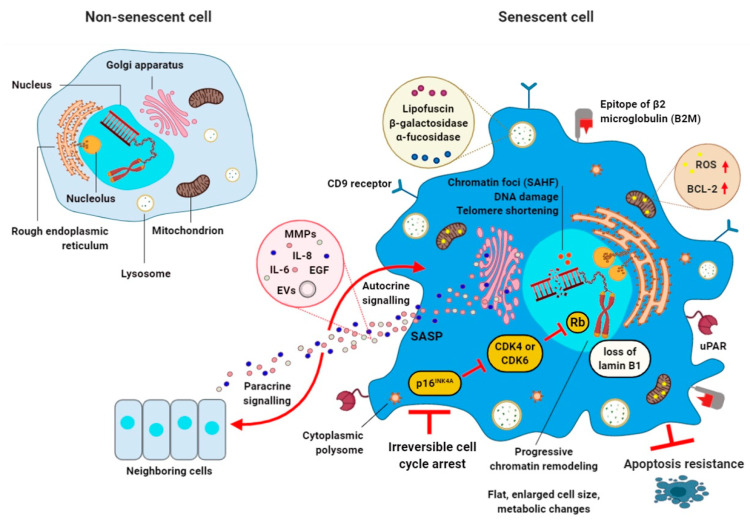
Biomarkers of cellular senescence. Senescent cell (**right**) is characterized by irreversible cell cycle arrest induced by various stressors (e.g., DNA damage or telomere shortening). Classical features of senescent cells include flat and enlarged cell size, elevated expression of cell cycle inhibitors (such as p16^INK4A^ or p21^Cip1^), increased nucleus size and multiple nucleoli, lysosomes overexpressing β-galactosidase, α-fucosidase, and lipofuscin, chromatin reorganization based on senescence-associated heterochromatic foci (SAHF) and loss of lamin B1, senescence-associated ribosome biogenesis defects (e.g., “free” cytoplasmic polysomes with ribosomes), the overexpression of anti-apoptotic Bcl-2 family members, reactive oxygen species (ROS) production in mitochondria, and expression of cell surface markers such as CD9 receptor, urokinase-type plasminogen activator receptor (uPAR), and epitope of β2 microglobulin (B2M). A characteristic key feature of cellular senescence is also the manifestation of the senescence-associated secretory phenotype (SASP) via the Golgi apparatus. SASP mediates the autocrine/paracrine activities of senescent cells by the secretion of matrix metalloproteinases (MMPs) such as MMP-3 and MMP-9, growth factors (e.g., epidermal growth factor (EGF)), cytokines and chemokines (e.g., IL-6 and IL-8), miRNAs, activins, and inhibins, lipids (e.g., ceramides), as well as exosome-like small extracellular vesicles (EVs). SASP can modify the microenvironment of senescent cells (SCs) and directly affects neighboring cells. A non-senescent cell is also presented (**left**).

SCs display common features such as granularity, increased activity of lysosomal senescence-associated β-galactosidase (SA-β-gal), lipofuscin accumulation, exclusion of proliferative markers, formation of senescence-associated heterochromatin foci (SAHF), and persistent expression of proteins that participate in DDR (Figure 1) [23,24,25]. Moreover, SCs are characterized by senescence-associated secretory phenotype (SASP) [26,27]. The SASP entails secretion of numerous molecules, such as inflammatory chemokines (e.g., MCP-1, MIP 1α, and CCL-16) and cytokines (e.g., IL-6 and IL-8), growth factors (e.g., IGF-1 and EGF) and angiogenic factors/regulators, miRNAs, proteases, and damage-associated molecular patterns (DAMPs), which can induce changes in neighboring cells via both paracrine and autocrine mechanisms [28,29,30,31]. SASP may have both beneficial and pathological functions. First, these factors are suggested to regulate immune clearance of SCs to prevent fibrosis and promote tissue regeneration [1,32,33]. Contrariwise, SASP factors can induce the development of secondary senescence within non-senescent nearby cells [34]. In addition, the SASP is responsible for promotion of low-grade chronic inflammation. This malfunction can alter the tissue microenvironment, which can also stimulate neoplastic cell growth, tumor metastasis, and angiogenesis [35].

A recently-proposed strategy to delay symptoms of aging is the suppression of SASP components [36]. Notably, compounds that interfere with the pathways involved in SASP regulation are the most effective. These include nuclear factor (NF)-κB, Janus kinase (JAK)/signal transducer and activator of transcription (STAT), mitogen-activated protein kinase (MAPK), and mammalian target of rapamycin (mTOR) pathways [37]. For example, various drugs have been shown to lower production or secretion of SASP factors. Simvastatin decreases production of interleukins IL-6 and IL-8 in vitro [38]. Natural compounds such as flavonoids have also been documented to impair the secretory phenotype in SCs [39]. Importantly, SASP is not the only mechanism by which SCs modulate their neighboring cells. For example, it has been shown that OIS is accompanied by a dynamic fluctuation of NOTCH1 activity [40], reactive oxygen species (ROS) production [41], or by release of exosomes [42]. Apart from irreversible withdrawal from the cell cycle and SASP, another feature of SCs is their resistance to apoptosis through the multilevel regulated pro-survival senescent cell anti-apoptotic pathways (SCAPs) such as p53/p21/serpins, BCL-2/Bcl-X_L_, PI3K/AKT/ceramide signaling, the hypoxia-inducible factor (HIF-1α) pathway, or HSP90-dependent networks [24,30,43].

## 2. Nano-Based Delivery Systems for Diagnostic and Therapeutic Purposes

**Figure 2:**
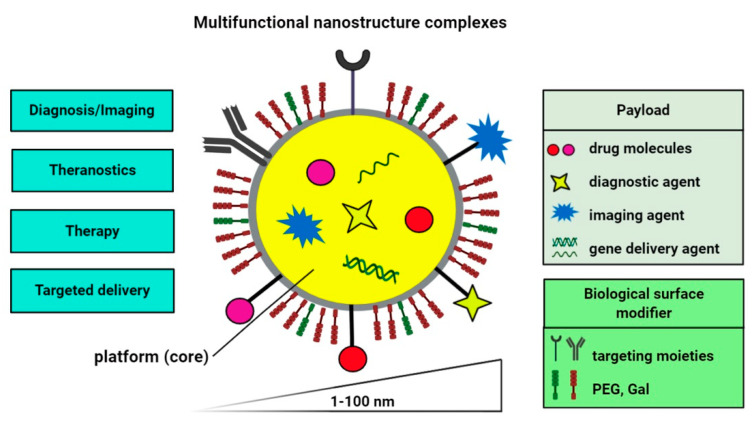
Schematic illustration of nanostructure-based multifunctional complexes. Cargos such as diagnostic probes (e.g., fluorescent or chromogenic dyes, radiotracers, or contrast agents), therapeutic agents (e.g., drugs, siRNA, natural compounds, or chemotherapeutics), surface coating (e.g., PEG, Gal, or mesoporous silica) and targeting ligands (e.g., antibodies or aptamers) can be attached/encapsulated to nanostructures using chemistry and surface modification methods. PEG, polyethylene glycol; Gal, galacto-oligosaccharides.

The applications of nanotechnology to medicine provide an opportunity to improve the safety, efficiency, and sensitivity of conventional medical therapeutics [44]. A nano-based drug delivery system relies on the use of nanostructures and nanomaterials for targeted transport of a therapeutic or diagnostic molecule and for its release in controlled manner [45,46]. In addition, the use of large-size structures in drug delivery is challenging, because of their poor bioavailability and stability, undesirable effects, limited targeted drug delivery, and therapy efficiency [47]. Recently, several approaches to drug delivery have been proposed to minimize these limitations by the development and production of smart nanomaterials. Nanomaterials have attracted widespread attention due to their unique properties such as size (100 nanometers or smaller in at least one dimension), shape, surface area, permeability, and various mechanical, magnetic, optical, and electronic properties [48,49,50,51]. Nanomaterial properties are significant in determining their important pharmacokinetics criteria, such as adsorption, distribution, accumulation, cellular uptake, and excretion mechanisms [52]. Uptake of nano-sized materials by cellular systems may occur via four different basic endocytic mechanisms, namely phagocytosis, macropinocytosis, clathrin-mediated endocytosis, and caveolae-mediated endocytosis [53]. Among the most-often-used nano-sized materials are nanoparticles (NPs), polymers, dendrimers, micelles, liposomes, carbon nanotubes, and fullerenes [52,54]. Moreover, an interesting idea in nanomedical approaches may be the design of multifunctional nanomaterial complexes able to carry out intracellular delivery of one or several cargos, such as diagnostic, imaging, or therapeutic molecules to specific locations in the body (e.g., cells, tissues, or organs) (Figure 2). Such conjugates, upon synthesis, coating, and functionalization, can integrate various functionalities [52,55]. Of note, surface functionalization and coating of nanostructures with various substances such as polymers [56,57], antibodies [58], peptides [59], or surfactants [60] may improve their biocompatibility and limit their immunogenicity. Polyethylene glycol (PEG)-coating of nanostructures (PEGylation) is one of the commonly-used chemical modifications, which may also affect the bioavailability of nano-sized materials [61]. The combination of imaging and therapeutic compounds within a single nanostructure complex may yield a theranostic nanodevice for improved diagnosis and therapy of various diseases such as cancer [62,63]. In addition, nanoformulations for cancer theranostics have been used for targeted drug delivery to solid tumors at relatively high concentrations with minimal toxic effects on surrounding normal cells or tissues [64,65].

The use of a nano-based drug delivery system is a rapidly-developing branch of nanomedicine where materials in the nanoscale range are employed to serve as diagnostic and therapeutic tools that may offer multiple benefits in treating human diseases [66]. One of the uses of nanocarriers as drug delivery systems is nano-encapsulation, conjugation, and site-specific transport of therapeutic agents or natural substances for therapeutic purposes [46]. For example, the nucleolin-targeting AS1411 aptamer-decorated niosomes can both effectively recognize cancer cells and deliver the nucleolipidic anticancer drugs that in turn promote antiproliferative effects against human cervical cancer cells [67]. Moreover, the efficiency of natural bioactive compounds, such as quercetin, curcumin, resveratrol, and berberine has been significantly improved by the use of nano-based drug delivery systems [46,68,69,70,71,72]. The properties of nanoscale-sized materials also often differ significantly from those of the same materials at large scales. Hence, nanotechnology has been used to improve the uptake of small agents [46,52]. Moreover, the small size of these agents may enable them to overcome various anatomical and physiological barriers which may improve nanodrug mobility and diffusivity to specific body parts [46,73]. For example, it has been shown that transferrin-containing gold nanoparticles have increased ability to cross the blood–brain barrier through transcytosis in vivo [74]. In addition, nanosized materials loaded with poorly-water-soluble and chemically-unstable compounds such as quercetin may improve their bioavailability and promote targeted delivery [75]. For example, a six-fold increase in bioavailability of thymoquinone (bioactive compound isolated from *Nigella sativa*) has been shown after its encapsulation in a lipid nanocarrier in comparison with the unmodified plant-derived compound. The combined use of lipid nanocarrier with thymoquinone increased the pharmacokinetic properties of the bioactive product resulting in better therapeutic effects [76]. Nanotechnology-based drug delivery systems may also protect drugs from the degradation in the gastrointestinal tract and bypass the liver first-pass metabolism, increasing lymphatic absorption and improving drug solubility. Moreover, drugs encapsulated in nano-structures have prolonged circulation time, reduced toxicity, decreased immunogenicity, and increased efficacy [46,77,78].

**Figure 3:**
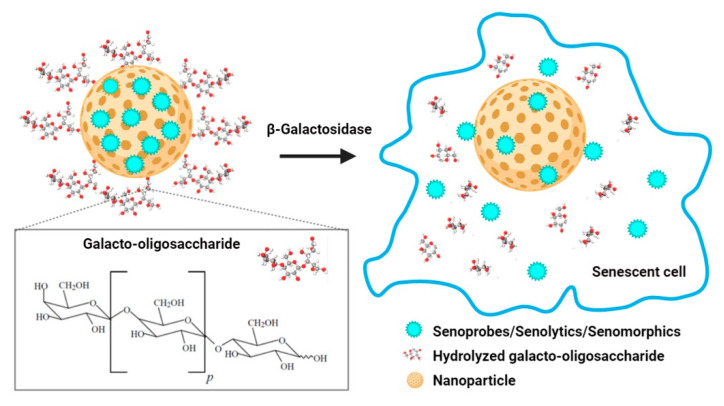
Nanoparticles capped with galacto-oligosaccharide and loaded with senoprobes, senolytics, and/or senomorphics. Cargos can be preferentially released and activated in senescent cells with high activity of SA-β-galactosidase that is based on SA-β-galactosidase-mediated hydrolysis of an oligosaccharide coat.

It is important to mention that stimuli-responsive nanocarriers (also called stimuli-triggered drug delivery) that provide a drug in a time-dependent and concentration-controlled manner can be sensitive to microenvironmental changes and various parameters, namely enzyme concentration, hypoxia, pH, heat, light or electronic pulses, magnetism, temperature and others [79]. For instance, stable at physiological pH, pH-responsive nanocarriers are promising agents for specific drug delivery to acidic diseased tissues, such as cancer [80]. pH-sensitive controlled release of doxorubicin from doxorubicin-encapsulated nanoparticles has been revealed to result in apoptosis in MCF-7 breast-cancer cells [81]. pH-sensitive nanoparticles loaded with the chemotherapeutic agent are also considered as non-toxic to normal tissues [82]. Recently, several nanocarriers have been proposed for efficient targeting of SCs in vitro and in vivo [83]. The most frequently-used method to detect SCs in vitro and in vivo is the evaluation of the senescence-associated β-galactosidase activity [84]. It was shown that nanoparticles coated with a substrate for SA-β-gal and encapsulated with cytotoxic drugs, small molecules, or probes were able to target cargo release mediated by increased SA-β-gal activity in SCs (Figure 3) [83,85]. Molecules that have been developed and designed to detect and visualize SCs are called senoprobes [55]. Importantly, the coated nanostructures are activated only in SCs, where the coating is digested and the cytotoxic drugs/senolytics can be released into the cytoplasm to induce apoptotic cell death that promotes healthspan and extends lifespan in mouse aging models [83,86].

## 3. Senolytics and Senotherapy

As cellular senescence is an important contributor to aging and age-related disorders, new approaches are required to postpone/prevent detrimental effects mediated by dysfunctional SCs. It has been shown that the selective elimination of SCs is able to attenuate numerous aging-related disorders and extend mouse longevity [87,88]. For instance, in progeroid BubR1 (BubR1^H/H^) mouse model, the selective eradication of SCs reduces age-related disorders, in a process termed senolysis [88]. It has been shown that removing senescent cells from tissues might inhibit cancer formation in mice suggesting that senolysis in older patients may have healthspan benefits [87]. Senolytic drugs and novel galacto-oligosaccharide-coated nanomaterials with toxic cargos can be considered to target SCs. Senolytic compounds are able to selectively kill SCs, whereas senomorphic compounds are able to diminish SASP [15,43,88,89]. It should be pointed out that senolytics must be precisely targeted to eliminate SCs without affecting normal neighboring cells [30].

Bioinformatics approaches based on RNA and protein expression profiles of senescent versus non-senescent cells have been used for identification of SCAPs. Key pro-survival proteins were recognized as potential targets for senotherapy. It is important to note that inhibition of pro-survival pathways and a decrease in the expression of SCAPs may promote death of SCs [43,90,91,92]. Selected drugs and small molecules from chemical libraries were found to interfere with key components of pro-survival pathways in senescent cells and were considered as first-generation senolytics [24]. Until now, several classes of first-generation senolytics such as natural compounds (e.g., fisetin, quercetin, and piperlongumine), a pan-receptor tyrosine kinase inhibitor dasatinib, HSP90 inhibitors (e.g., geldanamycin, 17-AAG, and 17-DMAG), and Bcl-2 family inhibitors (e.g., navitoclax, ABT-737, A1331852, and UBX1967) have been described [24,43,86,90,91,92,93,94,95,96,97,98,99]. Targeting SCAPs in culture in vitro using senolytics may result in apoptosis of some SCs. For example, it has been documented that a combination of drugs with senolytic activity, namely dasatinib (a tyrosine kinase inhibitor) and quercetin (a flavonoid with estrogenic activity) eliminates SCs in human and murine cultured cells in vitro and in vivo [90,93]. In addition, this combined treatment significantly improves physiological function and increases lifespan in prematurely-aged mice [90,94,95,96]. Another agent having senotherapeutic activity is a naturally-occurring flavonoid with low toxicity, fisetin. It has been shown that treatment of old and progeroid mice with fisetin reduces some senescence markers in multiple tissues and extends animal lifespan [97,98]. Moreover, fisetin preferentially induces apoptosis in senescent human umbilical vein endothelial cells [97]. It has been shown that a natural product, piperlongumine is also a senolytic agent and preferentially induces apoptosis in oncogene-induced senescent human fibroblast cells [99]. In addition, the senolytic potential of curcumin analog EF24 (a synthetic analog of natural anti-aging compound) in vitro has been reported that was achieved by apoptosis induction in SCs in an ROS-independent manner, and enhanced proteasome mediated-degradation of the Bcl-2 anti-apoptotic protein [100]. Panobinostat, a histone deacetylase inhibitor, also has post-chemotherapy senolytic activity in senescent cancer cells [101]. It has been shown that panobinostat is able to increase caspase 3/7 activity and decrease the expression of anti-apoptotic proteins leading to clearance of SCs, which accumulate after anticancer drug treatment. Another approach to removing SCs from aged mice is targeting the pathways involved in senescence such as the FOXO–p53 axis. Activated FOXO4 transcription factor can induce cellular senescence by interacting with p53, which leads to upregulation of the transcription of a major regulator of senescence, p21. A novel FOXO4-interacting peptide disturbs the FOXO4 communication with p53 and also can induce apoptosis in both prematurely- and naturally-aging mice. Treatment with FOXO4-peptide can enhance health and lifespan in old mice [102]. Recently, several compounds targeting the chaperone protein HSP90, namely 17-AAG (tanespimycin), geldanamycin, and 17-DMAG (alvespimycin) have been demonstrated to be effective in inducing apoptosis in senescent murine and human cells [43,103]. Interestingly, the first in-human pilot study (14 patients) demonstrated that dasatinib and quercetin can eliminate SCs in patients with age-related chronic lung disease (idiopathic pulmonary fibrosis), reduce inflammation, and limit frailty [93]. More recently, senolytic chimeric antigen receptor (CAR) T cells were designed and their efficacy in eliminating of SCs expressing urokinase-type plasminogen activator receptor (uPAR) in vitro and in vivo systems was documented [104]. It has been shown that uPAR-specific CAR T cells may preferentially eradicate SCs in a number of experimental models of senescence [104]. Moreover, BET-family protein degrader (BETd) has been tested for a potential senolytic activity in obesity-induced hepatocellular carcinoma mouse model [105]. It has been reported that BETd may induce senolysis by targeting non-homologous end joining (NHEJ) and autophagy [105]. Autophagy is generally considered as a protective and adaptive stress response, but in some cellular settings, autophagy may also contribute to cell death [106]. Indeed, BETd provoked autophagy-induced apoptosis in senescent cells that was achieved, at least in part, by up-regulation of autophagic gene expression [105].

Several other strategies have been recently proposed to provide more accurate and targeted senolytic-based interventions. These include nanotechnology-based approaches for detection, site-specific delivery of senolytics and/or senoprobes, and successful elimination of SCs [83,107,108,109,110]. As cellular senescence may be implicated in the pathogenesis of age-related neurodegenerative diseases such as Alzheimer’s disease and Parkinson’s disease [111,112], the use of nano-senolytics in the central nervous system (CNS) seems promising. One should remember that nano-based systems are able to overcome the blood–brain barrier and improve the delivery of encapsulated therapeutic agents and dietary polyphenols at lower systemic doses [68,74].

## 4. Nanomaterials for the Clearance of Senescent Cells

**Figure 4:**
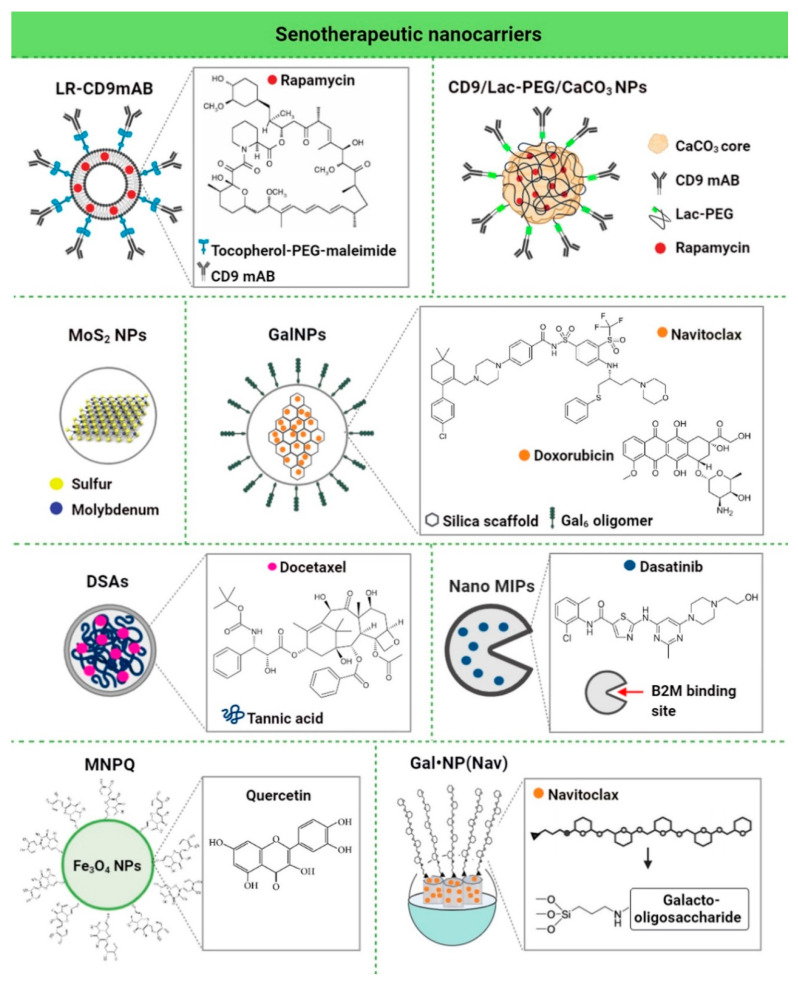
Different examples of nanocarriers used for targeted delivery of senolytics/senomorphics to senescent cells. Chemical structures of the loaded cargos are presented. LR, rapamycin-loaded PEGylated liposomes; NPs, nanoparticles; PEG, polyethylene glycol; Lac-PEG, lactose-polyethylene glycol; Gal, galacto-oligosaccharide; DSAs, docetaxel-tannic acid self-assemblies; NanoMIPs, molecularly imprinted nanoparticles; B2M, beta-2 microglobulin; Gal_6_, 6-mer galacto-oligosaccharide; Nav, navitoclax; MNPQ, quercetin surface-functionalized Fe_3_O_4_ nanoparticles.

As the main purpose of senotherapy is to kill SCs, safe and effective detection and targeting of these cells is crucial to improving human health and prolonging lifespan [113]. Nano-based systems developed to identify and kill senescent cells can be considered as second-generation targeted and selective senolytics that are able to efficiently eliminate senescent cells upon systemic administration without causing adverse side effects. One of the best-explored groups of nano-senolytics is smart nanodevices that are based on porous calcium carbonate nanoparticles, mesoporous silica nanoparticles, carbon quantum dots, and molecularly-imprinted polymer nanoparticles (nanoMIPs) (Figure 4) [114]. Targeted delivery and detection/elimination of SCs can be achieved by encapsulation of senolytics/senomorphics/fluorophores using a number of nanomaterials. For example, cargo release in the presence of β-galactosidase (β-gal) was due to the hydrolysis of the capping galacto-oligosaccharide (Gal) polymer [109]. In vitro studies demonstrated that nanomaterials covered with Gal and loaded with fluorophores (e.g., rhodamine B, indocyanine green, coumarin-6, or Nile blue) were preferentially activated in β-galactosidase-overexpressing SCs, which were able to lyse the galacto-oligosaccharide coat (Figure 3) [85,114]. Moreover, β-galactosidase-instructed supramolecular assemblies can also lead to the formulation of hydrogels and nanofibers in SCs, which decreases the expression of senescence-driving proteins [115]. Apart from β-gal, increased expression of other lysosomal hydrolases (e.g., α-L-fucosidase) has been used for detection of senescent cells [116]. To date, a collection of senoprobes has been described [114]. Nano-based senoprobes could be utilized to monitor the response of tumors to the administration of senescence-inducing chemotherapeutic drugs. More recently, another method for the real-time in vivo detection of senescent cells based on mesoporous silica nanoparticles loaded with Nile blue and coated with a galacto-hexasaccharide was proposed [117]. Functionalized nanomaterials appear to have a promising potential as nanocarriers and can be used for improving SC clearance. Table 1 provides published examples of in vitro and in vivo senotherapeutic action of nano-based drug delivery systems [83,107,108,109,110,114,118,119,120,121].

The first-reported SC-targeted cargo delivery nano-system is a nano-structure based on mesoporous silica nanoparticles (MSN S1) coated with galacto-oligosaccharides and loaded with rhodamine B [85]. MSN S1 are engulfed by human SCs and activated by SA-β-gal. There are also studies showing that encapsulation of a senolytic agent, namely navitoclax with β(1,4)-galacto-oligosaccharides is effective in clearing SCs in models of damage-induced and chemotherapy-induced senescence [109]. Nanostructures conjugated with drugs may also exert a senomorphic effect by inhibiting the SASP [107,120]. A recent in vitro study demonstrated that nanoparticles functionalized with monoclonal antibody against CD9 receptor (overexpressed in old cells) and loaded with rapamycin (an mTOR inhibitor with well-recognized anti-aging activity) (CD9-Lac/CaCO_3_/Rapa NPs) have anti-senescence effect [107]. CD9-Lac/CaCO_3_/Rapa NPs significantly improved the proliferative capacity of SCs, which was accompanied by lower expression of IL-6 and IL-1β, the SASP components [107]. Moreover, CD9-targeted PEGylated liposomes have been documented as a promising drug delivery platform to target senescent cells [118]. The uptake of CD9-targeted liposomes by premature senescent human dermal fibroblasts (HDFs) was revealed to be higher than that by young HDFs [118]. Targeted delivery of rapamycin (LR-CD9mAb) to diminish senescence of CD9-receptor-overexpressing cells was also explored and rapamycin was found to promote cell proliferation and reduce the levels of SA-β-gal-positive cells [118]. Another study performed by Ke et al. [108] showed that MoS_2_ NPs suppressed hydrogen-peroxide-induced senescence in endothelial cells. In addition, MoS_2_ NP treatment leads to autophagy activation, which suppressed SIPS in vitro [108]. More recently, the docetaxel–tannic acid self-assembly (DSA)-based nanoparticle strategy has been developed and documented to be an efficient delivery system to provide docetaxel to prostate cancer cells and xenograft tumors and diminish cellular senescence [119]. DSAs-induced anti-senescence and anti-cancer effects were mediated by the inhibition of TGFβR1/FOXO1/p21-associated senescence and apoptosis induction in vitro and in vivo [119]. The authors concluded that DSAs can be considered as a useful nano-platform to deliver anticancer molecules (here docetaxel) to cancer cells that would result in improved therapeutic benefits and limited chemotherapy-induced senescence and drug resistance [119]. Targeted clearance of SCs using B2M (β2 microglobulin) nanoMIPs (molecularly imprinted nanoparticles) has been also described [110]. B2M nanoMIPs specifically recognize one of the previously-identified members of a cell membrane surface protein that is overexpressed in SCs, namely B2M [110]. In addition, it has been shown that B2M nanoMIPs loaded with a senolytic drug (dasatinib) can kill senescent bladder cancer cells [110]. Moreover, fluorescently-tagged nanoMIPs detected senescent cells in vivo and were not toxic when injected at single doses [110]. The authors concluded that nanoMIPs may have diagnostic, prognostic, and therapeutic potential when considering the pathological conditions mediated by the accumulation of senescent cells [110]. We have also documented a senolytic and senostatic activity of quercetin surface-functionalized magnetite nanoparticles (MNPQ) in prematurely-senescent human fibroblasts (hydrogen peroxide treatment in vitro) [120]. It has been shown that MNPQ are able to eliminate senescent human fibroblast cells in vitro [120]. MNPQ diminish senescence-mediated proinflammatory response as judged by decreased secretion of IL-8 and IFN-β that was accompanied by the activation of AMP-activated protein kinase (AMPK) [120].

## 5. Conclusions

The nano-based drug delivery system and its applications in the field of aging research have developed and evolved over the last several years. Because of their nano-sized nature and their ability to meet diverse functionalities, senescent-cell-targeted nanocarriers may have potential for elimination of senescent cells from the human body, which may improve the treatment of age-related disorders. However, many challenges restrain their successful clinical translation. Firstly, our current knowledge about molecular mechanisms underlying cellular senescence and modulating senescence-associated secretory phenotype in the context of age-associated disorders is not yet complete. In addition, successful application of senotherapeutic nanocarriers may depend on the development of best-suited methods for minimizing potential off-target effects and maximizing on-target effects. Next, for those who study senescent-cell-targeted nanocarriers, it may be challenging to find an optimal nanocarrier type for senolytic drug delivery as well as optimal biocompatible concentration(s), improved drug encapsulation, and ligand conjugation efficiency. The parameters of nanocarrier absorption, interactions with human biological fluids, distribution, metabolism, and excretion are also important for future anti-aging applications of senotherapeutic nanocarriers. In conclusion, a nano-based drug delivery system constitutes a novel strategy to target senescent cells for potential therapeutic interventions. While few initial studies are promising, additional further studies involving the development of second-generation targeted and selective senolytics, namely senescence-targeted nanocarriers and validation of nano-senolytics using preclinical aging model systems and clinical trials are needed to determine whether these nanodevices might be effective against age-related diseases.

## Figures and Tables

**Table 1:** Nanomaterials targeting senescent cells using cellular in vitro and in vivo models.

Nanomaterial	Biologically- Active Component	Concentration	Senescence Model	In Vitro/In Vivo Model	Senolytic Effects and Mechanism	Ref.
LR-CD9mAb CD9 monoclonal antibody conjugated to PEGylated liposomes	Rapa	25 nM	Human dermal fibroblasts (HDF), doxorubicin-induced senescence	In vitro	Anti-senescence activity (increased proliferative potential, decreased β-galactosidase activity and p53/p21 expression, and increased cell migration)	[118]
CD9-Lac/CaCO_3_/Rapa NPs CD9 monoclonal antibody-conjugated lactose-wrapped calcium carbonate nanoparticles loaded with rapamycin	Rapa	0.2 mg of rapamycin per mg of CaCO_3_ NPs	Human dermal fibroblasts (HDF), replicative and doxorubicin-induced senescence	In vitro	Anti-senescence activity (decreased β-galactosidase activity and p53/p21/CD9/cyclin D1 expression, increased cell proliferation and cell migration ability, decreased population doubling time, and prevention of G1 cell cycle arrest)	[107]
MoS_2_ NPs molybdenum disulfide mesoporous silica nanoparticles	-	50 μg/mL	Human aortic endothelial cells (HAECs), stress-induced premature senescence	In vitro	Anti-senescence activity (decreased γ−H2AX phosphorylation, repressed upregulation of p16, p21 and p53, activation of autophagy, improved autophagic flux, and prevention of lysosomal and mitochondrial dysfunction)	[108]
GalNP(dox) 6-mer galacto-oligosaccharide encapsulated doxorubicin	Dox	1 mg/kg	Mouse, bleomycin-induced lung fibrosis	In vivo	Anti-senescence activity (improved lung function)	[109]
GalNP(nav) 6-mer galacto-oligosaccharide encapsulated navitoclax	Nav	0.06 mg/mL	Melanoma (SK-MEL-103), palbociclib-induced senescence	In vitro	Senolytic activity (apoptosis of senescent cells)	
GalNP(dox) 6-mer galacto-oligosaccharide encapsulated doxorubicin	Dox	1 mg/kg	Mouse-bearing SK-MEL-103 tumor xenografts, palbociclib-induced tumor senescence	In vivo	Clearance of senescent cells and induced regression of tumor xenografts	
GalNP(nav) 6-mer galacto-oligosaccharide encapsulated navitoclax	Nav					
DSAs Docetaxel-tannic acid self-assemblies (DSAs)-based nanoparticles	Docetaxel	2.5-5 nM	Prostate cancer cells (C4-2 and PC- 3)	In vitro	Senolytic activity (inhibition of senescence-related TGFβR1, FOXO1, and p21 proteins and activation of apoptosis)	[119]
		30 mg/kg	Mouse-bearing PC-3 tumor xenografts	In vivo	Clearance of senescent cells (induced regression of tumor xenografts by blockade of TGFβR1/p21-mediated senescence signaling and activation of apoptosis)	
NanoMIPs molecularly-imprinted nanoparticles	Dasatinib	10 μM dasatinib-conjugated B2M nanoMIPs	Bladder cancer cells with a tetracycline (tet)-regulatable p16 expression systems (EJp16)	In vitro	Senolytic activity (decreased number of senescent cancer cells)	[110]
MNPQ quercetin surface-functionalized Fe_3_O_4_ nanoparticles	Quercetin	5 μg/mL	Human foreskin fibroblasts (BJ), hydrogen peroxide-induced senescence	In vitro	Senolytic and senostatic activity (AMPK activation, induction of non-apoptotic cell death, and inhibition of SASP components, namely IL-6 and IFN-β)	[120]
GalNP(nav)	Nav	40 mg GalNP (Nav)/kg (≈2.5 mg/kg of free navitoclax)	Triple-negative breast cancer mouse model, palbociclib-induced senescence	In vivo	Senolytic activity (inhibited tumor growth, reduced metastasis, and limited systemic toxicity of navitoclax, and apoptosis of senescent cancer cells)	[121]

Abbreviations: Rapa, rapamycin; Dox, doxorubicin; Nav, navitoclax.

## Abbreviations

17-AAG	tanespimycin
17-DMAG	alvespimycin
AKT	protein kinase B
AMPK	AMP-activated protein kinase
B2M	Beta-2 microglobulin
Bcl-2	B cell lymphoma 2 family
BETd	BET family protein degrader
CAR	Chimeric antigen receptor
CCL-16	Monotactin-1
CDKI	Cyclin-dependent kinase inhibitor
CS	Cellular senescence
DAMPs	Damage-associated molecular patterns
DDR	DNA damage response
DNA	Deoxyribonucleic acid
Dox	Doxorubicin
DSAs	Docetaxel-tannic acid self-assemblies
EGF	Endothelial growth factor
EGF	Epidermal growth factor
EVs	Extracellular vesicles
FOXO4	Forkhead box protein O4
Gal	Galacto-oligosaccharide
Gal_6_	6-mer galacto-oligosaccharide
HAECs	Human aortic endothelial cells
HDF	Human dermal fibroblasts
HIF-1α	Hypoxia inducible factor 1α
HSP90	Chaperone heat shock protein 90
IFN-β	Interferon beta
IGF-1	Insulin-like growth factor 1
IL-6	Interleukin 6
IL-8	Interleukin 8
IL-1β	Interleukin 1β
JAK	Janus kinase
Lac-PEG	Lactose-polyethylene glycol
LR	Rapamycin-loaded PEGylated liposomes
MAPK	Mitogen-activated protein kinase
MCP-1	Monocyte chemoattractant protein 1
MIP-1α	Macrophage inflammatory protein 1α
miRNA	MicroRNA
MMPs	Metalloproteinases
MoS_2_	Molybdenum disulfide
MNPQ	Quercetin surface-functionalized Fe_3_O_4_ nanoparticles
MSN	Mesoporous silica nanoparticles
mTOR	Mammalian target of rapamycin
nanoMIPs	Molecularly-imprinted polymer nanoparticles
Nav	Navitoclax
NF	Nuclear factor
NHEJ	Non-homologous end joining
NPs	Nanoparticles
OIS	Oncogene-induced senescence
p16	Cyclin-dependent kinase inhibitor 2A
p21	Cyclin-dependent kinase inhibitor 1A
p53	Tumor suppressor protein p53
PEG	Polyethylene glycol
PI3K	Phosphatidylinositol-3-kinase
Rapa	Rapamycin
Rb	Retinoblastoma
RNA	Ribonucleic acid
ROS	Reactive oxygen species
SAHF	Senescence-associated heterochromatin foci
SASP	Senescence-associated secretory phenotype
SA-β-gal	Senescence associated β-galactosidase
SCAPs	Senescent cell anti-apoptotic pathways
SCs	Senescent cells
SIPS	Stress-induced premature senescence
siRNA	Small interfering RNA
STAT	Signal transducer and activator of transcription
uPAR	Urokinase-type plasminogen activator receptor
